# Circulating HMGB1 is elevated in veterans with Gulf War Illness and triggers the persistent pro-inflammatory microglia phenotype in male C57Bl/6J mice

**DOI:** 10.1038/s41398-021-01517-1

**Published:** 2021-07-12

**Authors:** Carla Garza-Lombó, Morrent Thang, Hendrik J. Greve, Christen L. Mumaw, Evan J. Messenger, Chandrama Ahmed, Emily Quinn, Kimberly Sullivan, Michelle L. Block

**Affiliations:** 1 Department of Pharmacology and Toxicology, The Stark Neurosciences Research Institute, Indiana University School of Medicine, Indianapolis, IN USA; 2 Department of Biostatistics, Boston University School of Public Health, Boston, MA USA; 3 Department of Environmental Health, Boston University School of Public Health, Boston, MA USA; 4 Roudebush Veterans Affairs Medical Center, Indianapolis, IN USA

**Keywords:** Molecular neuroscience, Diseases

## Abstract

Gulf War Illness (GWI) is a chronic, multi-symptom peripheral and CNS condition with persistent microglial dysregulation, but the mechanisms driving the continuous neuroimmune pathology are poorly understood. The alarmin HMGB1 is an autocrine and paracrine pro-inflammatory signal, but the role of circulating HMGB1 in persistent neuroinflammation and GWI remains largely unknown. Using the LPS model of the persistent microglial pro-inflammatory response, male C57Bl/6J mice injected with LPS (5 mg/kg IP) exhibited persistent changes in microglia morphology and elevated pro-inflammatory markers in the hippocampus, cortex, and midbrain 7 days after LPS injection, while the peripheral immune response had resolved. Ex vivo serum analysis revealed an augmented pro-inflammatory response to LPS when microglia cells were cultured with the 7-day LPS serum, indicating the presence of bioactive circulating factors that prime the microglial pro-inflammatory response. Elevated circulating HMGB1 levels were identified in the mouse serum 7 days after LPS administration and in the serum of veterans with GWI. Tail vein injection of rHMGB1 in male C57Bl/6 J mice elevated TNFα mRNA levels in the liver, hippocampus, and cortex, demonstrating HMGB1-induced peripheral and CNS effects. Microglia isolated at 7 days after LPS injection revealed a unique transcriptional profile of 17 genes when compared to the acute 3 H LPS response, 6 of which were also upregulated in the midbrain by rHMGB1, highlighting a distinct signature of the persistent pro-inflammatory microglia phenotype. These findings indicate that circulating HMGB1 is elevated in GWI, regulates the microglial neuroimmune response, and drives chronic neuroinflammation that persists long after the initial instigating peripheral stimulus.

## Introduction

Gulf War Illness (GWI) is a chronic multi-symptom illness with chronic central nervous system (CNS) and peripheral symptoms and pathology [1–5]. Behavioral deficits and functional brain changes have been reported in veterans with GWI, where a broad range of brain regions are affected, including the hippocampus, basal ganglia, white matter, and cortex [6–11]. Increasing evidence implicates perturbation of the peripheral immune system in both peripheral and CNS symptoms [12–26], where distinct differences in GWI-associated circulating factors are beginning to be identified [25, 27, 28]. Importantly, a recent report documented the elevation of the [(11)C]PBR28 PET signal, in areas including precuneus, prefrontal, primary motor and somatosensory cortices, demonstrating a persistent response from microglia, the resident myeloid cells in the CNS parenchyma, in veterans with GWI, long after the theater of war [29].

Microglia readily respond to events in the periphery [30], such as systemic lipopolysaccharide (LPS) administration [31], intestinal ischemia-reperfusion injury [32], liver damage [33], pulmonary injury with ozone [34], and many others. Importantly, the microglial and neuroimmune response to peripheral injury has been shown to augment ongoing neuropathology [32, 34–36], highlighting the potential importance of peripheral damage and immune dysfunction on CNS effects, particularly as a possible source of pathology in GWI.

Accumulating reports indicate that microglia have complex, function-specific phenotypes [37], where microglial dysregulation is implicated in progressive neuronal damage in diverse CNS conditions and disease [30, 38]. In fact, microglia can be persistently dysregulated to drive pathology long after the instigating stimulus, such as the case with LPS [31] or chemotherapy [39], but little is understood about this persistent phenotype. The neuroimmune hypothesis of GWI [25, 27] holds that this persistent neuroinflammation, the chronic elevation of cytokines and reactive oxygen species in the brain that persists long after the instigating stimulus during the Gulf War, drives the associated CNS neuropathology and behavioral deficits. Several murine GWI models have exhibited microglial phenotype shifts and neuroinflammation [5, 40–42].

High-mobility group box 1 (HMGB1), a ubiquitously expressed non-histone DNA binding protein, is actively secreted by immune cells and by damaged cells as an alarmin to function as an autocrine and paracrine signal and cytokine [43]. HMGB1 disrupts the blood-brain barrier [44] and activates cells of myeloid lineage, where HMGB1 can initiate a pro-inflammatory response in microglia, macrophages, and monocytes [45]. Peripheral HMGB1 has been linked to CNS effects, as intraperitoneal (IP) injection of HMGB1 decreases performance on memory tasks in mice [46] and induces depressive-like behavior acting as a late-phase inflammation mediator [47, 48]. Consistent with this, several studies show HMGB1 release after systemic LPS injection [49–52], but the function of circulating HMGB1 is controversial. HMGB1 acts as a common mediator of chronic inflammatory diseases [43], where it has been found in circulation during many CNS diseases [53, 54]. Importantly, rats exposed to GWI-related chemicals pyridostigmine bromide (PB), DEET, and permethrin, in combination with moderate stress as a GWI model exhibited elevated extracellular vesicles containing HMGB1 in the brain and serum, supporting a potential role for circulating HMGB1 in GWI [55]. However, the specific role of circulating HMGB1 in the persistent microglial neuroinflammation response and GWI is largely unknown.

At present, there is significant mechanistic uncertainty regarding how immune dysfunction in GWI could culminate in persistent microglial responses that persist years later. To begin to address this question and start to define the basic biology of the persistent pro-inflammatory microglial (PPM) response, we employed the LPS persistent neuroinflammation model and serum from GWI veterans to explore whether: (1) Microglia fundamentally change and have a unique transcriptional signature during persistent neuroinflammation; (2) Circulating factors, like HMGB1, regulate the PPM phenotype; (3) HMGB1 is elevated in the serum of veterans with GWI when compared with healthy control veterans.

## Materials and methods

### Animals

C57BL/6 male mice (6–8 weeks old) were obtained from Jackson Laboratories (Bar Harbor, ME, USA). Animals were housed in an Association for Assessment and Accreditation of Laboratory Animal Care (AAALAC)—official housing facility and maintained at 20–24 °C on a 12 H light–dark cycle Mice were individually housed in HEPA-filtered, ventilated polycarbonate cages (Lab Products, Inc., Seaford, DE) and were provided an NTP-2000 diet (Harlan Laboratories) and water ad libitum. Mice were acclimated for 1 week after delivery. All experiments were conducted with the International Care and Use Committee (IACUC) approval and the Guidelines for the Care and Use of Laboratory Animals (National Institutes of Health, Bethesda, MD, USA) were followed.

### LPS persistent neuroinflammation model

Mice received a single IP injection of LPS (strain O111:B4, EMD Millipore Chemicals, Lot number 3388502, 6 EU/ng, 5 mg/kg) or vehicle (0.9% saline), which is an established murine model of persistent neuroinflammation that continues across the lifespan of the mouse [31, 56], where LPS is unable to enter the brain parenchyma [31, 56]. The single instigating LPS dose causes peripheral inflammation and the elevation of circulating cytokines that rapidly triggers an immediate microglial pro-inflammatory response [57, 58]. While the initiating circulating pro-inflammatory response of the LPS administration is complete in the periphery by 24H after LPS administration [31], the microglial response persists for at least 1 week after the LPS treatment (LPS persistent neuroinflammation model) and can continue across the animal’s lifetime to eventually culminate in behavioral deficits and neuropathology in several GWI regions, including the substantia nigra (midbrain) and hippocampus months after treatment [31, 56, 58]. While the exact pathobiology of GWI is unknown, the LPS persistent neuroinflammation model was selected because of the: brain regions affected similar to GWI [8–11], whole-body immune perturbation similar to GWI [12–25], and chronic neuroimmune consequences that persist long after the instigating stimulus, mimicking GWI [29].

Mice were humanely anesthetized with isoflurane and euthanized by exsanguination via cardiac puncture. Samples were collected at either 3 H (acute response) or 7 days (persistent response) following LPS injection, as previously reported [57]. One cohort of mice was euthanized and microglia were isolated from the whole brain. A second cohort of mice was euthanized and the right hemisphere of each brain was fixed in a 4% paraformaldehyde solution and cryopreserved in 30% sucrose, while the left hemisphere was dissected into GWI-affected regions (hippocampus, cortex, and midbrain) and snap frozen. The serum, liver, and spleen were also collected and either processed immediately or stored at −80 °C. Further details are specified in the Supplementary Information.

### HMGB1 and TLR4 small molecule inhibitors

Mice were treated with small molecule HMGB1 and Toll-like receptor (TLR) 4 inhibitors after the initiation of the LPS pro-inflammatory response to address their ability to ameliorate the persistent response [31] As an inhibitor of HMGB1 secretion, Inflachromene (10 mg/kg) [59] or vehicle (distilled water containing 5% DMSO and 40% polyethylene glycol) was administered IP daily for 6 days, beginning 24 H after LPS injection, after the initial instigating peripheral circulating cytokine response has resolved [31]. TLR4 is a primary receptor for HMGB1 on immune cells [60]. As such, TLR4 receptor antagonist TAK-242 (3 mg/kg) [61] or vehicle (PBS containing 0.9% DMSO) was also administered IP for 6 days, commencing 24 H after LPS injection.

### Intravenous (IV) recombinant HMGB1 protein administration

Previous reports indicate that approximately 20–50 μg of circulating HMGB1 can accumulate in circulation after LPS treatment in mice [62]. Mice received a single intravenous (IV) injection of carrier-free recombinant mouse HMGB1 protein (rHMGB1, 32.5 μg in 200 μl) or vehicle (20 mM Tris-HCl, pH 8.0, 0.2 M NaCl, 1 mM DTT) (Thermo Scientific-Invitrogen, Carlsbad, CA, USA). At 3 H following injection, the right hemisphere of each brain was fixed in a 4% paraformaldehyde solution and cryopreserved in 30% sucrose, the left hemisphere was dissected into the hippocampus, cortex, and midbrain. The liver and spleen were also collected and either processed immediately or stored at −80 °C.

### Whole-brain adult microglia isolation

At 3 H and 7 days post-injection, mice were anesthetized and perfused with 50 mL cold phosphate-buffered saline (PBS). Adult microglia were isolated from whole adult brains, as previously described [57, 63]. Further details are specified in the Supplementary Information.

### IBA1 immunohistochemistry

Chromogenic microglial IBA1 histochemistry was performed as previously described [57, 63]. Further details are specified in the Supplementary Information.

### Microglia morphology quantification

To assess changes in cell volume, images of IBA1 and fluorescent staining in the hippocampus (dentate gyrus and CA1) were acquired as Z-stacks (1 μm steps) using a Nikon A1R Confocal microscope and a 40X objective (Nikon Instruments, Melville, NY). Seven hippocampus slices were analyzed per mouse. Hypertrophic microglia were defined as cells with a volume greater than 500 μm^3^ and counted using the NIS Elements program (Nikon Instruments, Melville, NY).

### Enzyme-linked immunosorbent assay (ELISA)

TNFα and HMGB1 protein were assessed in the serum with commercial ELISA kits from R&D Systems (Minneapolis, MN, USA) and Tecan IBL International (Hamburg, Germany), according to the manufacturer’s instructions.

### Immunology multiplex assay

Cytokine protein levels were determined using a Milliplex^®^ assay kit (MAP Mouse Cytokine/Chemokine Magnetic Bead Panel—Immunology Multiplex Assay, #MCYTOMAG-70K Merck-Millipore, Burlington, MA, USA) following the manufacturer’s instructions.

### BV2: microglial cell line

BV2 mouse microglia cell lines were acquired from NIEHS/NIH in 2002 from the John Hong lab and have been repeatedly validated in the past [64] and present. Cultures are tested for mycoplasma and the BV2 cells are negative. BV2s were cultured at 37 °C in High-Glucose Dulbecco’s Modified Eagle Medium (DMEM) supplemented with 10% Fetal Bovine Serum (FBS), 50 U/mL penicillin, and 50 μg/mL streptomycin in a humidified incubator with 5% CO_2_/ 95% air.

### Quantitative reverse transcription polymerase chain reaction (RT-qPCR)

Total RNA was extracted from brain regions, liver, and spleen tissues with TRIzol (Thermo Scientific-Invitrogen, Carlsbad, CA, USA), following the manufacturer’s instructions. RT-qPCR was performed as previously described [57, 63]. Further details are specified in the Supplementary Information.

### Nanostring gene expression analysis

RNA was extracted from the isolated microglia with a Qiagen’s RNeasy Micro kit (Hilden, Germany) with on-column DNase digestion following the manufacturer’s instructions. Then, 50 ng of RNA for each sample was processed using the nCounter Mouse Neuroinflammation Panel (Seattle, WA, USA), gene expression analysis was performed on the nCounter system (NanoString Technologies), and analyzed using nSolver analysis software (NanoString Technologies) according to the manufacturer’s instructions. Further details are specified in the Supplementary Information.

### Gulf war veteran serum samples

Serum samples from 80 Gulf War veterans (40 GWI cases, 40 healthy GW veteran controls) from the DOD funded Boston Gulf War Illness Consortium (GWIC) biorepository were used for this study. All participants provided written informed consent to participate in the study. This study was reviewed and approved by the Boston University and Indiana University institutional review board. Further details are specified in the Supplementary Information.

### Statistical analysis

The sample size was determined based on prior reports. A randomized block design was employed for the animal experiments and sample processing was performed blind, where the code denoting treatment groups was only provided for data analysis. Data are expressed as the mean ± SEM. The ROUT method in GraphPad Prism was used to identify outliers, *Q* = 1. The Levine test was used to assess the homogeneity of variance, where p > 0.05 indicated homogeneity. Data were analyzed by analysis of variance (ANOVA) using GraphPad Prism (GraphPad Prism, San Diego, CA, USA) when the variance was homogenous. When the assumption of homogeneity of variance was not met, the Kruskal–Wallis test was used, followed by post-tests. Mean differences were analyzed by Bonferroni’s post-hoc analysis or Student’s *t* test, when appropriate. A *p*-value < 0.05 was considered statistically significant. We also conducted statistical comparisons of GWI cases vs. controls comparing serum HMGB1 levels, self-reported pyridostigmine bromide (anti-nerve agent) use, using linear regression models adjusting for age and sex [65]. The least squares means with standard error (LSM ± SE) are reported.

## Results

### Persistent pro-inflammatory microglia (PPM) responses without traditional circulating factors

**Fig. 1:**
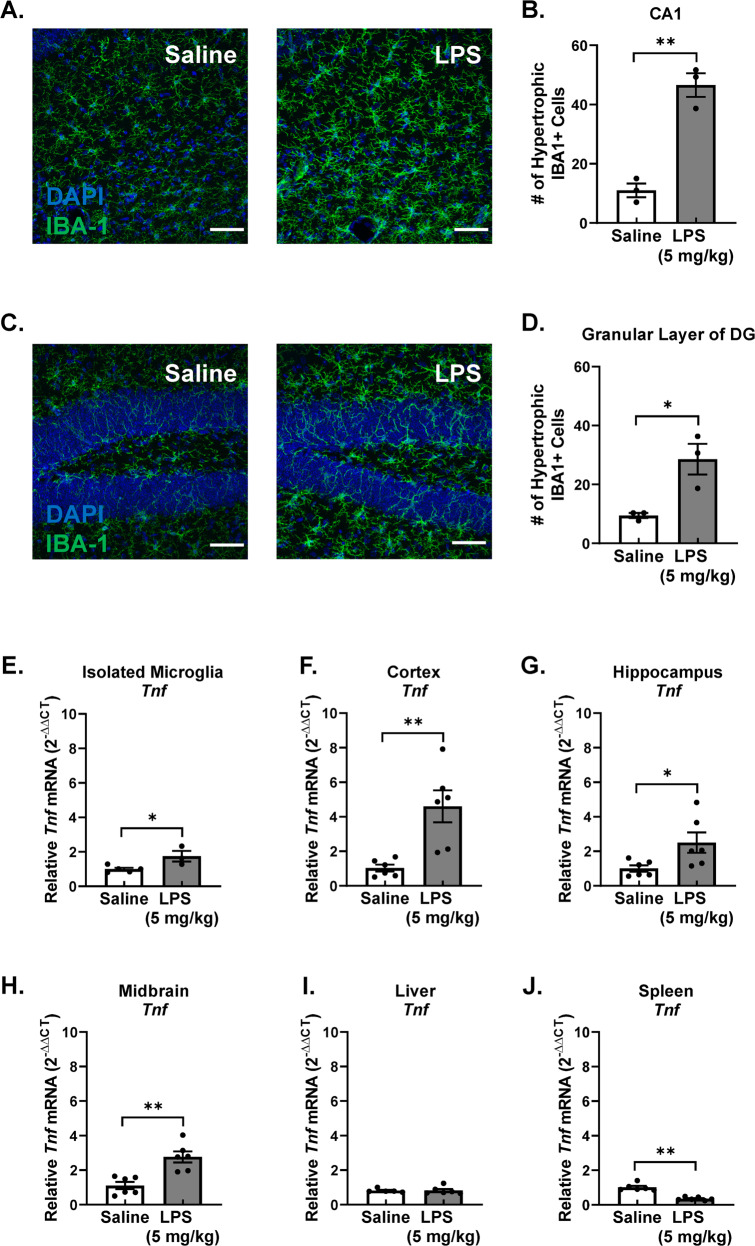
The persistent pro-inflammatory microglia response 7 days after LPS administration in a Murine GWI-like model. Male C57BL/6 mice received a single IP injection of LPS or saline. Microglia morphology and neuroinflammation were assessed 7 days later in this model of Gulf War Illness—like neuroimmune responses that persist long after the instigating stimulus. Immunofluorescent assessment of microglia morphology changes in the (**A**) CA1 region and (**C**) the granular layer of the DG was performed, where representative maximum intensity projection images taken at ×40 are shown, the scale bar depicts 50 μm, and IBA1 (green) and DAPI (blue) are stained. The number of hypertrophic IBA1 + microglia cells were counted in the (**B**) CA1, and (**D**) granular layer of the DG. Hypertrophic cell = volume > 500 μm^3^. Data are reported as the mean ± SEM. *p < 0.05 and **p < 0.01 when compared to control (*n* = 3). To determine the source of the pro-inflammatory response, *Tnf* (TNFα) mRNA levels were measured in the (**E**) isolated microglia, (**F**) cortex (**G**) hippocampus, (**H**) midbrain, (**I**) liver, and (**J**) spleen by qRT-PCR and normalized to *Gapdh* using the 2-ΔΔCT method. Data are reported as the mean ± SEM. *p < 0.05 and **p < 0.01 when compared to control (*n* = 3–6).

Circulating pro-inflammatory factors have been established as critical mediators in the initial transfer of the cytokine response in the periphery to the brain, but the identity of those that could be driving persistent neuroinflammation after this instigating stimulus is unknown. To begin to address this question, we first confirmed persistent changes in microglia morphology in the hippocampus 7 days after LPS administration, as evidenced by increased numbers of microglia with larger cell volume (hypertrophic microglia, larger than 500 μm^3^) in the CA1 region, the granular layer of the dentate gyrus and the subgranular layer of the dentate gyrus, (Fig. 1A–D and Supplementary Fig. 1A, B, *p* < 0.05). Increased mRNA elevation of the prototypical pro-inflammatory cytokine TNFα was also confirmed in several brain regions affected by GWI, such as the cortex, hippocampus, and midbrain, where isolated microglia from the whole brain also showed *Tnf* mRNA upregulation, demonstrating that microglia themselves are contributors to the persistent neuroinflammation in brain regions affected by GWI (Fig. 1E–H, *p* < 0.05). These data indicate that persistent neuroinflammation occurs in brain regions that are affected in GWI 7 days after LPS-administration and that microglia themselves are a source of the persistent pro-inflammatory response, which continues long after the instigating stimulus.

**Fig. 2:**
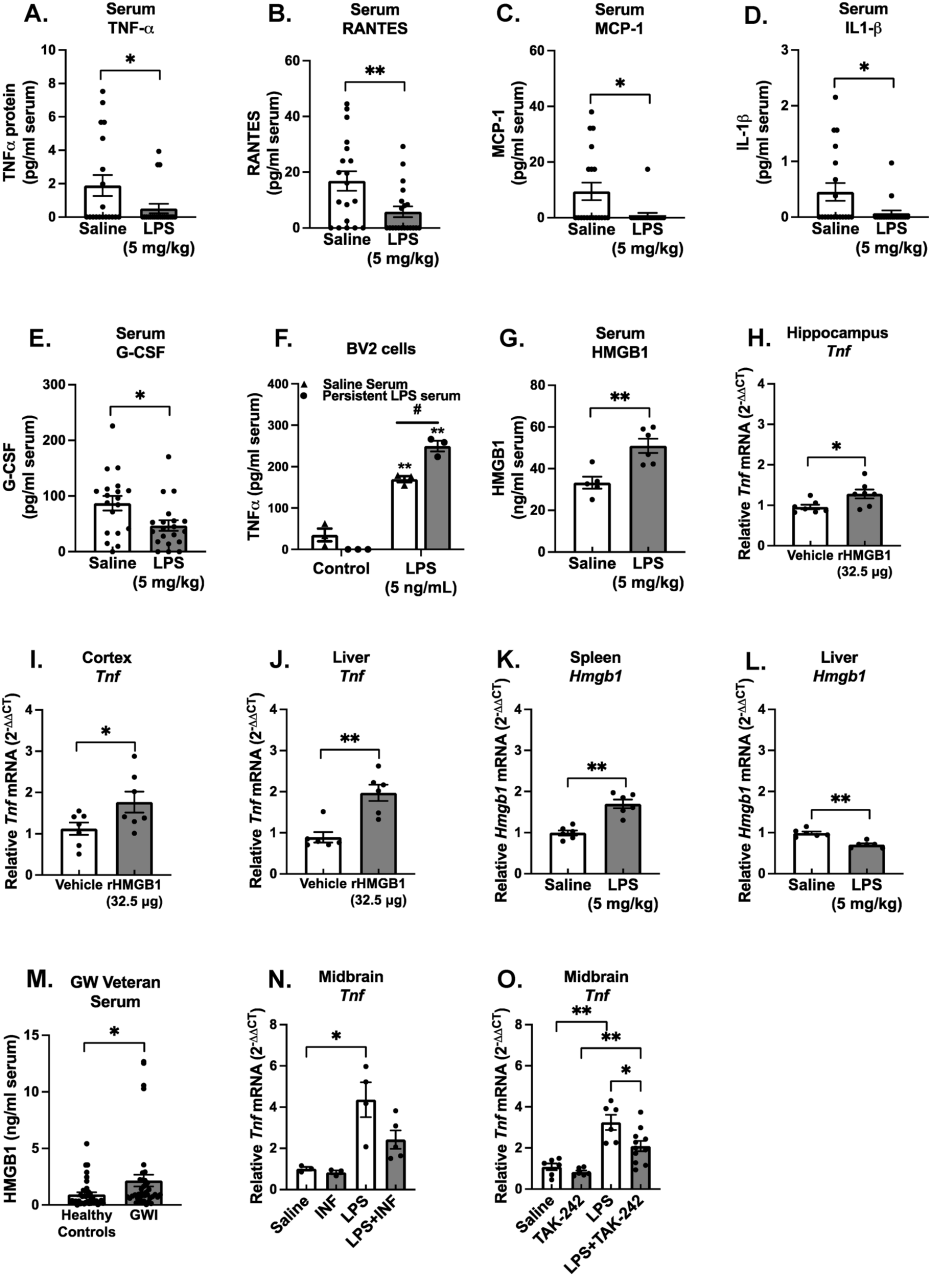
The LPS PPM mouse model serum has ex vivo neuroimmune bioactivity, pro-inflammatory factors below baseline, and elevated HMGB1: implications for Gulf War Illness. Male C57BL/6 mice received a single IP injection of LPS or saline and the serum was collected 7 days later in this model of Gulf War Illness- like persistent neuroinflammation. Protein levels of circulating traditional pro-inflammatory factors were determined in the serum using the Milliplex^®^ multiplex assay and ELISA: (**A**) TNFα, (**B**) RANTES, (**C**) MCP-1, (**D**) IL1-β and (**E**) G-CSF, (**G**) HMGB1. (**F**) To assess neuroimmune bioactivity ex vivo, BV2 mouse microglia cells were treated with 2% serum obtained 7 days following LPS or saline injection with media alone or LPS (5 ng/ml), where the supernatant TNFα was measured 3 H later by ELISA. Data are reported as the mean ± SEM. *p < 0.05 and **p < 0.01 when compared to control and ^#^*p* < 0.05 when compared between exposure groups (*n* = 5–20). *Hmgb1* mRNA was assessed 7 days after LPS injection in the, (**K**) spleen *Hmgb1* mRNA, and (**L**) liver. To determine if circulating HMGB1 could elicit neuroinflammation, C57BL/6 mice received a single tail vein injection of rHMGB1 or vehicle and *Tnf* mRNA expression was assessed 3 H later in the (**H**) hippocampus, (**I**) cortex, and (**J**) liver. mRNA levels were analyzed by qRT-PCR and normalized to *Gapdh* using the 2-ΔΔCT method. Data are reported as the mean ± SEM. *p < 0.05 and **p < 0.01 when compared to control (*n* = 3–7). (**M**) Serum samples from 40 veterans with Gulf War Illness (GWI) that had met the Kansas diagnostic for GWI and 40 healthy controls were assessed for levels of circulating HMGB1 by ELISA, Data are reported as the mean ± SEM. *p < 0.05 when compared to control (*n* = 40). To determine if HMGB1 inhibition could abolish the LPS-induced persistent neuroinflammation, at 24H after LPS injection, C57BL/6 mice were administered (**N**) Inflachromene (HMGB1 inhibitor,10 mg/kg) or vehicle IP or (**O**) TAK-242 (3 mg/kg, TLR4 inhibitor) or vehicle for 6 days and *Tnf* mRNA expression was assessed in the midbrain. Data are reported as the mean ± SEM. *p < 0.05 and **p < 0.01 when compared to control (*n* = 3–12).

Serum, liver, and the spleen are important mediators of the instigating peripheral and CNS response to LPS [66–68], but little is known about their role in the persistent immune responses. The liver exhibited no significant difference in the *Tnf* mRNA response 7 days after LPS administration (Fig. 1I, *p* > 0.05) and the spleen showed a significantly attenuated TNFα response (Fig. 1J, *p* < 0.05), supporting that the peripheral immune response was resolved at 7 days after the LPS administration. While most serum pro-inflammatory factors were unchanged 7 days after LPS administration, TNFα, RANTES, MCP-1, IL-1β, and G-CSF were all present at significantly lower levels 7 days after LPS administration, when compared to saline-treated mice (Fig. 2A–E, *p* < 0.05). These findings indicate that at 7 days after LPS administration during the persistent microglial pro-inflammatory response, the peripheral pro-inflammatory response had resolved, including the lowering of traditional pro-inflammatory factors in circulation.

### Serum from the LPS persistent neuroinflammation model is bioactive

We next sought to determine whether an unknown factor was present in the serum that could regulate microglial function and neuroinflammation from the periphery. Using a previously established ex vivo serum bioactivity assay [69], BV2 microglia cells were pretreated with 2% of serum from saline-treated mice or serum from mice treated with LPS 7 days after the injection, when neuroinflammation was present in the absence of traditional pro-inflammatory factors in circulation. After 30 min, cells were then treated with either control media or 5 ng/mL LPS and supernatant was collected 3 H later and assessed for TNFα production. Data reveal that the 7 Day LPS serum did not instigate a TNFα response on its own, but significantly augmented the microglial response to LPS (Fig. 2F, *p* < 0.05), demonstrating that despite the absence of traditional pro-inflammatory factors, the serum primed BV2 microglia to an enhanced pro-inflammatory phenotype.

### Circulating HMGB1 is elevated in the LPS persistent neuroinflammation model, increased in veterans with GWI, and triggers neuroinflammation

We chose a hypothesis directed approach to begin to address what the pro-inflammatory targets in the serum might be, as HMGB1 is known to be: elevated in the serum of diverse CNS conditions [53, 54], and is increased in serum exosomes of a GWI mouse model [55]. We report here that the serum HMGB1 response remains elevated at 7 days after the LPS exposure in mice (Fig. 2G, *p* < 0.05) during the persistent neuroinflammation (Fig. 1, *p* < 0.05). Using a similar concentration to the amount that accumulates in the murine circulation after injection of LPS [62], tail vein injection of recombinant HMGB1 (32.5 μg) demonstrates that circulating HMGB1 triggers neuroinflammation 3 H later, as evidenced by elevated *Tnf* mRNA in the hippocampus and cortex (Fig. 2H, I*p* < 0.05). Interestingly, liver *Tnf* mRNA was significantly upregulated by rHMGB1 tail vein injection (Fig. 2J, *p* < 0.05), but spleen *Tnf* mRNA was not affected (Supplementary Fig. 2). *Hmgb1* mRNA expression was not altered in hippocampus, liver, and spleen (Supplementary Fig. 3A–C). Importantly, the serum from Gulf War Veterans diagnosed with GWI, as determined by the Kansas definition, exhibited significantly elevated serum HMGB1 levels as determined by ELISA, compared to healthy Gulf War Veteran controls (Fig. 2M, *p* < 0.05). There was a significant trend in the correlation between serum HMGB1 levels and self-reported exposure to pyridostigmine bromide, which was used as an prophylactic against sarin nerve gas (Supplementary Table S1, *p* = 0.0530), further supporting that circulating HMGB1 may be generalizable to GWI-specific exposures.

Notably, *Hmgb1* mRNA expression was not altered at 7 Days after LPS administration in the hippocampus, cortex, or midbrain (Supplementary Fig. 4A–C), supporting that brain parenchymal *Hmgb1* gene changes were not present in the transcriptional signature of the persistent neuroinflammation. Further, there was a significant decrease in liver *Hmgb1* mRNA levels (Fig. 2L, *p* < 0.05) and an increase in the spleen (Fig. 2K, *p* < 0.05) at 7 days after LPS administration, further highlighting the potential importance of the peripheral HMGB1 response in persistent neuroinflammation.

To further confirm that HMGB1 is regulating the persistent neuroimmune effects of LPS, we next used the small molecule inhibitors inflachromene and TAK-242, to inhibit HMGB1 secretion and activation of the HMGB1 receptor TLR4, respectively. Inhibition of HMGB1 secretion with inflachromene treatment reduced the *Tnf* expression comparable to control levels (Fig. 2N, *p* < 0.05) and TLR4 inhibition reduced the persistent neuroinflammation when compared to the LPS alone group (Fig. 2O, *p* < 0.05). These findings support that: circulating HMGB1: can trigger neuroinflammation; is elevated during LPS-induced persistent neuroinflammation 7 days after LPS administration; regulates the LPS persistent neuroinflammation response, and is increased in Gulf War veterans diagnosed with GWI.

### The persistent pro-inflammatory microglia (PPM) phenotype has a unique transcriptional signature

**Fig. 3:**
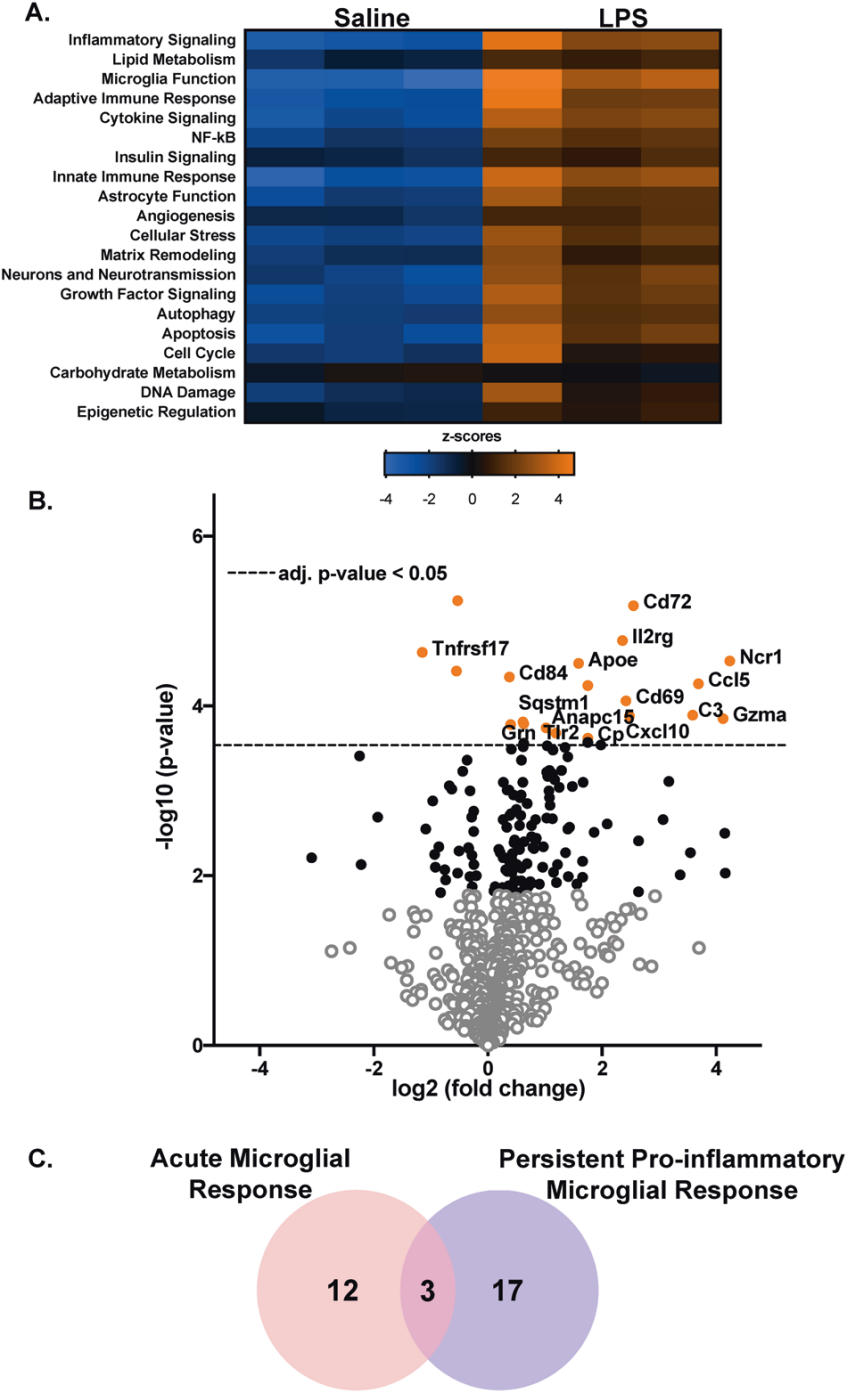
The persistent pro-inflammatory microglia (PPM) transcriptome. Male C57BL/6 mice received a single IP injection of LPS or saline and microglia were isolated from whole brains at 7 days later. The nanoString Mouse Neuroinflammation Panel was used to determine differential mRNA expression. (**A**) Hierarchical clustering of the differentially expressed genes was assessed and significantly enriched biological pathways were identified by Ncounter system. A heatmap depicting the gene expression by category is depicted for the saline and LPS treatment at 7 days after administration. Orange indicates high expression; blue indicates low expression. (**B**) A volcano plots depicting the differential mRNA expression in 7 days LPS vs Saline. Values shown are the log2 (fold change) ratios of LPS treatment with respect to the saline group plotted against the −log10 (*p*-value). The black dots denote the analyzed genes with *p*-value below the given False Discovery Rate (FDR) or *p*-value threshold. The horizontal line indicates the False Discovery Rate (FDR) threshold or adjusted p-value <0.05 determined by the Benjamini–Yekutieli and the orange dots indicate the most statistically significant genes (*p* < 0.05). (**C**) The Venn diagram illustrates the overlap of significantly modified genes by treatment (*n* = 3).

In an effort to define the unique transcriptional fingerprint of persistently activated microglia, we compared the distinct gene expression profiles of microglia isolated 3 H (acute response) and 1 week (persistent response) after LPS exposure. While gene expression changed at both 3 H (Supplementary Fig. 5A, B and Table S2) and 7 days. RNA isolated from microglia collected from mice 7 days after LPS administration demonstrated a significant, specific gene expression profile, when compared to the saline-treated group, where genes in all but the carbohydrate metabolism pathway were upregulated in the 7 days LPS group compared to the saline (Fig. 3A, *p* < 0.05). As expected, the pathways that scored higher on this list include Inflammatory Signaling, Microglia Function, Adaptive Immune Response, Cytokine Signaling, and NF-kB (Supplementary Table S3). The volcano plot (−log_10_ (*p* value) vs. fold change) in Fig. 3B shows genes differentially expressed in microglia between the saline and LPS treated mice at 7 days after treatment. The Venn diagram (Fig. 3C) illustrates that after correction, there were a total of 12 genes significantly altered by LPS treatment at 3 H, 20 genes were significantly changed by LPS at the 7 day time point, and 3 of those genes were shared between the two time points. Notably, the change in expression of only 17 genes could be attributed to only the 7 day persistent pro-inflammatory microglia phenotype. Supplementary Table S4 lists the genes significantly modified after correction at only 7 Days after LPS injection. Taken together, these data reveal that microglia during persistent neuroinflammation have a unique transcriptionally active phenotype.

**Fig. 4:**
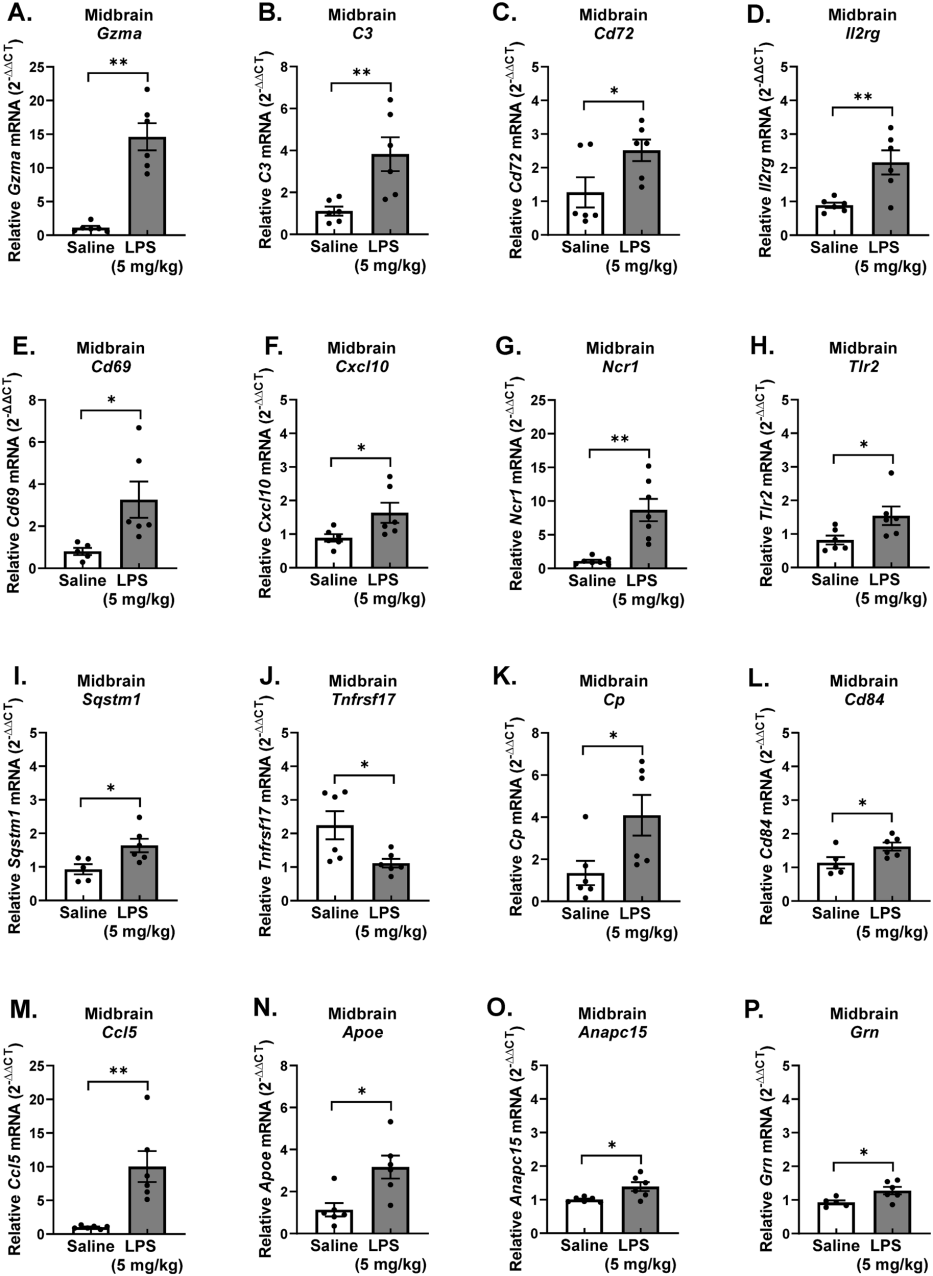
Persistent pro-inflammatory microglia (PPM) gene expression in the midbrain during persistent neuroinflammation. Male C57BL/6 mice received a single IP injection of LPS or saline and midbrain mRNA gene expression of PPM genes was assessed 7 days later. (**A**) *Gzma*, (**B**) *C3*, (**C**) *Cd72*, (**D**) *Il2rg*, (**E**) *Cd69*, (**F**) *Cxcl10*, (**G**) *Ncr1,* (**H**) *Tlr2*, (**I**)*Sqstm1*, (**J**) *Tnfrsf17,* (**K**) *Cp*, (**L**) *Cd84*, (**M**) *Ccl15*, (**N**) *Apoe*, (**O**) *Anapc15, and* (**P**) *Grn* mRNA levels were analyzed by qRT-PCR and normalized to *Gapdh* using the 2-ΔΔCT method. Data are reported as the mean ± SEM. *p < 0.05 and **p < 0.01 when compared to control (*n* = 4–6).

**Fig. 5:**
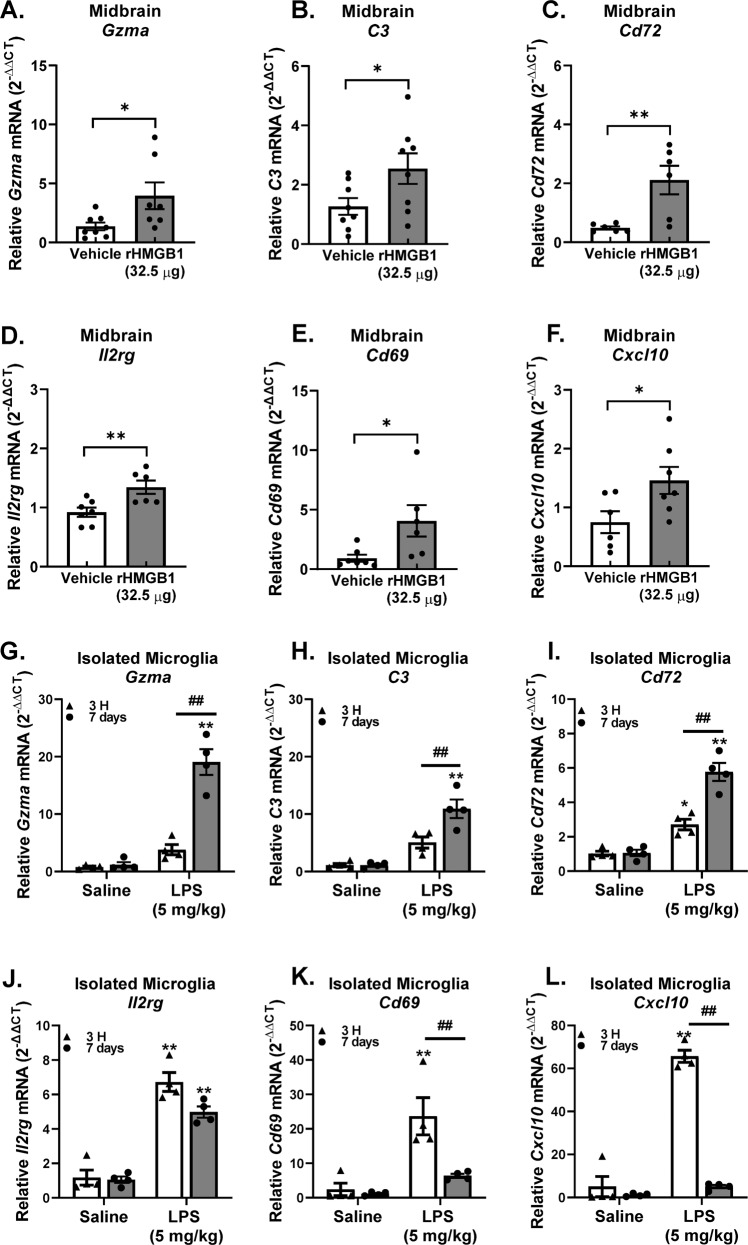
Intravenous HMGB1 administration elevates persistent pro-inflammatory microglia (PPM) genes in the midbrain. C57BL/6 mice received a single IV injection of recombinant HMGB1 protein (rHMGB1, 32.5 μg) or vehicle and mRNA was assessed in the midbrain 3 H later. PPM transcriptome genes (**A**) *Gzma*, (**B**) *C3*, (**C**) *Cd72*, (**D**) *Il2rg*, (**E**) *Cd69*, and (**F**) *Cxcl10* mRNA levels were measured in the midbrain. Male C57BL/6 mice received a single IP injection of LPS or saline and neuroinflammation was assessed 3 H or 7 days later and PPM gene expression levels were compared: (**G**) *Gzma*, (**H**) *C3*, (**I**) *Cd72*, (**J**) *Il2rg*, (**K**) *Cd69*, and (**L**) *Cxcl10* mRNA levels were measured in the isolated microglia. mRNA levels were analyzed by qRT-PCR and normalized to *Gapdh* using the 2-ΔΔCT method. Data are reported as the mean ± SEM. *p < 0.05 and **p < 0.01 when compared to control (*n* = 4–6).

Midbrain mRNA was used to confirm that the PPM cell gene expression changes occurred in the whole brain, as this GWI brain region showed an increase of *Tnf* gene expression and several prior reports have extensively characterized the midbrain neuroimmune pathology in LPS Persistent Neuroinflammation model [31, 56]. RT-qPCR analysis revealed significant changes in the following persistent microglial neuroinflammation genes in the midbrain in the 7 Day LPS Persistent Neuroinflammation model: *Gzma*, *C3*, *Cd72*, *Il*2rg, *Cd69*, *Cxcl10*, *Ncr1*, *Tlr2*, *Sqstm1*, *Tnfrsf17*, *Cp*, *Cd84*, *Ccl5*, *Apoe*, *Anapc15*, and *Grn* (Fig. 4A–O, *p* < 0.05). Moreover, RT-qPCR analysis also demonstrates that midbrain mRNA from rHMGB1 treated mice exhibited upregulation of a smaller subset: *Gzma*, *C3*, *Cd72*, *Il2rg*, *Cd69, and Cxcl10* (Fig. 5A–F, *p* < 0.05), demonstrating that circulating HMGB1 contributes to this transcriptional signature.

To further understand the unique PPM phenotype, the expression of the 6 PPM genes that rHMGB1 upregulates was compared in mRNA from isolated microglia from 3 H and 7 days after LPS treatment (Fig. 5G–L, *p* < 0.05). Notably, the *Il2rg*, *Cd69, Cxcl10* subset of genes were shown to peak at 3 H and persist at lower levels of expression at the 7 Day time point after LPS administration (Fig. 5J–L, *p* < 0.05). However, the second subset of genes *Gzma*, *C3*, and *Cd72* demonstrated a significantly higher expression level at the 7 Day time point, when compared to the acute response, 3 H after LPS administration (Fig. 5G–I, *p* < 0.05). These findings demonstrate that PPM cells have a unique transcriptome, rather than just a muted chronic pro-inflammatory response that continues over time. While some pro-inflammatory genes did peak at the acute time point shortly after LPS administration and continue at lower levels during the persistent response, another subset of PPM genes are most robustly upregulated at the 7-day time point, supporting that the PPM cells are fundamentally changed during the process of persistent neuroinflammation.

## Discussion

Peripheral immune dysfunction in GWI is hypothesized to contribute to persistent microglial neuroinflammation underlying the pathobiology years after the theater of war, but the mechanisms underlying how circulating factors can culminate in persistent neuroinflammation that continues long after the instigating stimulus has been largely unknown. Here, using the LPS model of persistent neuroinflammation, we confirm that microglia specifically contribute to the persistently elevated *Tnf* response in the brain, when serum was devoid of traditional pro-inflammatory circulating cytokines. Despite the absence of these traditional circulating triggers of neuroinflammation, the serum 7 days after LPS administration was bioactive regardless, augmenting the microglial pro-inflammatory response ex vivo. The alarmin HMGB1 was discovered to be elevated in the serum of both the murine LPS model of persistent neuroinflammation and in veterans with GWI, but *Hmgb1* gene expression changes were absent from the GWI-affected CNS brain regions and was instead limited to the periphery of the LPS model of persistent neuroinflammation. Mechanistically, intravenous rHMGB1 administration triggered neuroinflammation in brain regions that are affected in GWI. Microglia isolated from the LPS model of persistent neuroinflammation exhibited a unique transcriptional phenotype, where 6 of these genes were also upregulated by intravenous rHMGB1 administration. These findings provide much needed insight into how the periphery regulates the neuroimmune response outside of traditional circulating pro-inflammatory cytokines, identifies the transcriptional fingerprint of the PPM phenotype, and implicates circulating HMGB1 as one critical factor in the persistent microglia phenotype elevated in GWI.

Circulating cytokines are known to regulate neuroinflammation, changed circulating factors in GWI have been identified [25, 28, 70], and circulating factors are implicated as a potential mechanism in how peripheral GWI pathology could result in CNS effects. However, a recent study documented persistent microglial activation in veterans with GWI, as assessed by PET imaging, and showed no association of the microglial responses with the traditional pro-inflammatory circulating factors measured [71]. In the current study, using the LPS model of persistent neuroinflammation, we reveal an entire list of circulating traditional pro-inflammatory factors that are unnecessary for murine serum to be bioactive and regulate the microglia pro-inflammatory response (Fig. 2F). As such, these findings support that: PPM responses can be driven from the periphery, resolution of classic pro-inflammatory response in the periphery may not correlate with neuroinflammation or neuropathology, the traditional circulating cytokines may be misleading markers for persistent neuroinflammation, and ex vivo serum bioactivity assays with microglia may provide important insight into when peripheral pathology is regulating the neuroimmune response. Future studies should explore the feasibility of serum bioactivity assays as a potential diagnostic and therapeutic aid and also focus on the identification of other peripheral signals that can drive a PPM phenotype.

Circulating HMGB1 was identified as a mechanism of communication between the periphery and the brain to regulate the PPM response. Here, we show that the HMGB1 protein was in circulation long after the instigating single LPS IP injection (7 days, Fig. 2F), after the classic peripheral pro-inflammatory response had resolved (Fig. 2L) and during persistent neuroinflammation (Fig. 1). It is important to note that the post-translational modification of HMGB1, including redox state [72, 73], has a significant impact on receptor interactions driving the possible pro-inflammatory [74], chemotactic [75], or even anti-inflammatory [76] role of HMGB1, emphasizing the importance of the choice of inhibitor. Here, we note that inhibition of HMGB1 secretion and the TLR4 receptor ameliorated the persistent inflammatory effects of LPS (Fig. 2N, O), emphasizing its role in the persistent response. We also demonstrate that circulating HMGB1 is significantly elevated in veterans with GWI (Fig. 2M) and associated with self-reported pyridostigmine bromide exposure (Table S5). Further, data reveal that intravenous administration of rHMGB1 into circulation in mice is sufficient to trigger the neuroinflammation response (Fig. 2H–J), including a unique microglial pro-inflammatory transcriptome (Fig. 3). Interestingly, of all tissues tested, *Hmgb1* mRNA production was only shown to be elevated in the spleen at 7 days after LPS administration in the persistent neuroinflammation model (Fig. 2K), suggesting the periphery may be important for peripheral HMGB1 production in this model. However, at present, the peripheral source of circulating HMGB1 is unknown and should be a point of inquiry for future studies. Taken together, these findings point to circulating HMGB1 as a potential peripheral mechanism in the delayed and progressive CNS symptoms of GWI, particularly the persistent microglial response.

Accumulating evidence indicates that microglia express specific gene markers for multiple phenotypes including aging, disease, psychiatric disease, sex, viral infections, neurodegenerative diseases, and more [77]. Here, we explored whether the microglia were fundamentally changed during persistent neuroinflammation and not only do we demonstrate that these microglia exhibits a transcriptional profile 7 days after LPS administration that is unique from the acute 3 H response (Fig. 3), but that circulating HMGB1 by itself triggers the expression of 6 of these genes in the brain (*Gzma, C3, Cd72, Cxcl10, Cd69*, and *Il2rg*) (Fig. 5), where some of these factors have been associated with GWI and GWI-like symptoms. For example, the *C3* gene encodes for complement C3 and is not only a critical component of the complement system, but it is: activated in human AD brains, required for neurodegeneration in mouse models of amyloidosis and tauopathy [78], and has been previously shown to be upregulated in the cortex of a mice model of GWI [55]. CXCL10 (IP-10) is a small-molecular-weight pro-inflammatory chemokine produced by many cells, including microglia [79] and the CXCL10 protein was found upregulated in GWI serum and serum from myalgic encephalomyelitis patients [21]. While we found an increase in *Cxcl10* gene expression 7 days after LPS administration in the mouse model (Fig. 4F), we did not see any significant changes in the serum levels of the CXCL10 (IP-10) protein. We also show that *Cd72* is elevated in response to HMGB1-induced persistent neuroinflammation (Fig. 4C), where *Cd72* is a receptor expressed on microglia [80] and is a gene commonly upregulated in the brain after repeated LPS administration [81]. However, at present, there are no other reports testing the association of *Cd72* with GWI models nor GWI. In addition, *CD69* is type II C-lectin membrane bound receptor and a marker of lymphocyte activation [82] that is expressed in a subset of microglia in the CK-p25 inducible mouse model of severe neurodegeneration [83]. Although we found an increase in *Cd69* gene expression 7 days after LPS administration in the mouse model (Fig. 4E), its role in GWI has not yet been explored outside of the current study and its function in microglia is poorly understood. Interleukin 2 receptor gamma (*Il2rg*) is an important signaling component for many interleukin receptors expressed on microglia [71], where despite the upregulation in gene expression we show in response to persistent neuroinflammation (Fig. 4D) and HMGB1 (Fig. 5D), the implications for this CNS or peripheral immune gene expression in GWI are also unknown. Finally, while *Gzma*, the gene *for* Granzyme A, is expressed in myeloid cells [84], it’s expression in microglia specifically and role in GWI has yet to be explored. Importantly, examining the pattern of expression of these 6 PPM genes over time revealed that some PPM genes peaked early at the acute 3 H time point shortly after LPS administration and continued on at lower levels during the persistent response. However, another subset of PPM genes had the largest increase at the 7 day time point, demonstrating a unique transcriptional fingerprint for PPM cells. These findings may provide important insight into the mechanisms underlying persistent neuroinflammation that continues long after the initial stimulus and may provide much needed insight into potential GWI biomarkers and therapeutic targets.

**Fig. 6:**
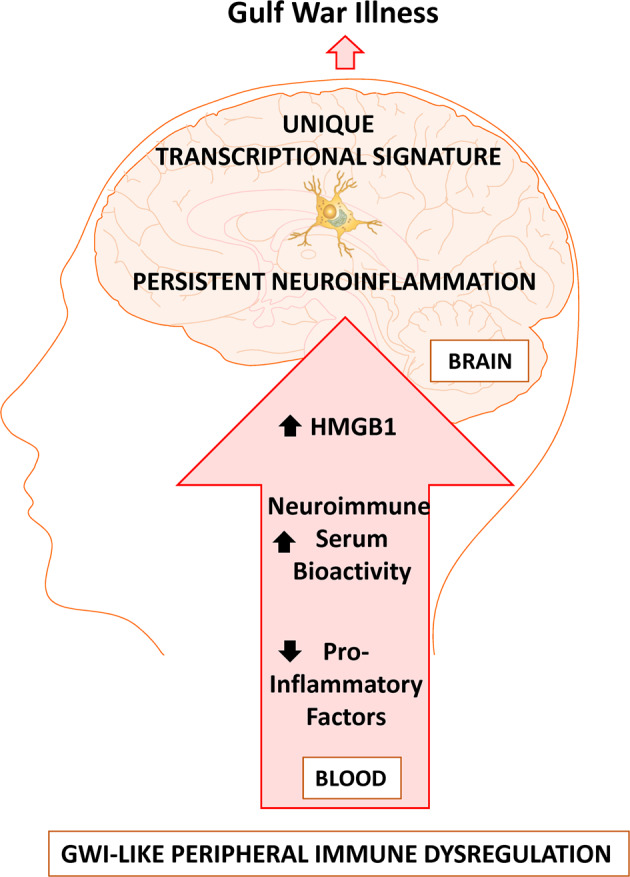
Circulating HMGB1 is elevated in Gulf War Illness and drives persistent neuroinflammation. Peripheral immune perturbation from IP LPS administration in the murine LPS Persistent Neuroinflammation model can mimic peripheral immune dysregulation similar to GWI that results in persistent neuroimmune bioactive serum that can modify the microglial pro-inflammatory response, long after the prototypical pro-inflammatory response in the serum has resolved. Inhibition of HMGB1 attenuates the persistent pro-inflammatory response in the brain and administration of recombinant HMGB1 can trigger neuroinflammation. Circulating HMGB1 is upregulated in the serum of Gulf War Veterans and in the bioactive serum of mice during the LPS-induced persistent neuroinflammation. Isolated microglia reveal a unique Persistent Pro-inflammatory Microglia transcriptome that is triggered in part by HMGB1, which may provide important insight into the persistent nature of neuroinflammation in GWI.

In summary, GWI presents as a whole body illness and how the periphery could regulate the persistent CNS neuroimmune response in GWI is poorly understood. In the current study, data show that after peripheral immune perturbation with LPS, circulating factors present in the serum, independent of traditional pro-inflammatory factors, have neuroimmune bioactivity and regulate the microglial response ex vivo. Data also reveal that HMGB1 is present in this LPS persistent neuroinflammation model 7 days after LPS administration, during the persistent neuroinflammation and after the peripheral immune response has resolved. Importantly, HMGB1 was also elevated in serum from Gulf War veterans diagnosed with GWI, compared to healthy controls, further supporting the importance of circulating HMGB1 for GWI. Tail vein injection of rHMGB1 in mice confirmed that circulating HMGB1 triggers neuroinflammation. Further analysis of microglia isolated from the LPS persistent neuroinflammation GWI-like model characterized the fundamental neuroimmune changes that occurred in these cells and identified a unique microglia transcriptome, where rHMGB1 tail vein injection also specifically elevated 6 of these genes (*Gzma, C3, Cd72, Cxcl10, Cd69*, and *Il2rg*) in the midbrain. Taken together, these findings point to circulating HMGB1 as a critical mechanism underlying how the periphery in GWI might regulate the persistent microglial response and identify novel potential biomarkers, unique therapeutic targets, and a serum bioactivity assay to elucidate the pathobiology and target future treatment in GWI (Fig. 6).

## Supplementary information

Supplemental Methods

Supplemental Figure Legends and Tables

Supplemental Figures

Nanostring Data-3H

Nanostring Data-7Day

## Data Availability

The datasets generated during and/or analyzed during the current study are available from the corresponding author on reasonable request and the Nanostring data is uploaded as a supplemental file.
