# Supplementary material for: Circulating HMGB1 is elevated in veterans with Gulf War Illness and triggers the persistent pro-inflammatory microglia phenotype in male C57Bl/6J mice
Source: Transl Psychiatry. 2021 Jul 12;11:390. doi: 10.1038/s41398-021-01517-1 (PMC8275600; doi:10.1038/s41398-021-01517-1)
Supplement: Supplementary file 1 — Supplemental Methods [file 41398_2021_1517_MOESM1_ESM.docx]

**SUPPLEMENTAL METHODS AND MATERIALS**

***Reagents***

LPS (strain O111:B4) was purchased from EMD Chemicals (Gibbstown, NJ, USA). The inhibitors TAK-242 and inflachromene were purchased from Merck Millipore (Darmstadt, Germany). Cell culture reagents were obtained from Thermo Scientific–Invitrogen (Carlsbad, CA, USA). The polyclonal antibody against the ionized calcium-binding adapter molecule 1 (IBA1) microglial marker was purchased from Wako (Richmond, VA, USA). The biotinylated goat anti-rabbit secondary antibody was purchased from Vector Laboratories (Burlingame, CA, USA). The secondary Alexa Fluor 488 goat anti-mouse IgG antibody was purchased from Thermo Scientific–Invitrogen and 4′,6-diamidino-2-phenylindole (DAPI) was purchased from Roche Diagnostics (Indianapolis, IN, USA). ELISA kits were purchased from R&D Systems (Minneapolis, MN, USA) and Tecan IBL International (Hamburg, Germany), all other reagents were acquired from Sigma-Aldrich (St. Louis, MO, USA).

***LPS Persistent Neuroinflammation Model***

The single instigating LPS dose causes peripheral inflammation and the elevation of circulating cytokines that rapidly triggers an immediate microglial pro-inflammatory response ^1, 2^. The microglial response persists for one week after the LPS treatment (LPS persistent neuroinflammation model) and can continue across the animal’s lifetime to eventually culminate in behavioral deficits and neuropathology in several GWI regions, including the substantia nigra (midbrain) and hippocampus months after treatment ^2-4^. While the exact pathobiology of GWI is unknown, the LPS persistent neuroinflammation model was selected because of the: GWI-like brain regions affected ^5-8^, whole body immune perturbation similar to GWI ^9-22^, and chronic neuroimmune consequences that persist long after the instigating stimulus, mimicking GWI ^23^. As such, to assess the role of the periphery on the persistent neuroinflammation phenotype, samples were collected at either 3 H (acute response) or 7 days (persistent response) following LPS injection, as previously reported ^1^.

***Whole Brain Adult Microglia Isolation***

Adult microglia were processed at 3 H and 7 days post-injection mice were anesthetized and perfused with 50 mL cold phosphate buffered saline (PBS). Whole brain tissue (400 mg) was diced with a sterile razor, suspended in 1 mL Hank’s buffered saline solution (HBSS) without CaCl_2_ and MgCl_2_ and centrifuged 2 min at 300g, 4 ºC. A single-cell suspension was prepared using the Miltenyi Neural Tissue Dissociation Kit (Miltenyi Biotec, San Diego, CA, USA) according to the manufacturer’s instructions. The single-cell suspension was washed with 10 mL HBSS containing CaCl_2_ and MgCl_2_ (Corning, Corning, NY, USA) and centrifuged for 10 min at 300g, 4 ºC. Next, the cells were depleted of myelin by suspension in 3 mL of 30% isotonic Percoll (GE Healthcare Life Sciences, Pittsburgh, PA, USA) followed by a 10 min centrifugation at 700g, 4 ºC. The cell pellet was washed in 5 mL HBSS without CaCl_2_ and MgCl_2_ and isolation of microglia was performed with magnetic CD11b microbeads (Miltenyi, San Diego, CA, USA) and MACS magnetic separator (Miltenyi, San Diego, CA, USA) following the manufacturer’s instructions.

***Quantitative Reverse Transcription Polymerase Chain Reaction (RT-qPCR)***

The RNA was treated with Ambion DNase I enzyme (Thermo Scientific-Invitrogen, Carlsbad, CA, USA) and reverse transcription of RNA (0.3 - 1.0 µg/sample) was performed with the Maxima First Strand cDNA Synthesis Kit (Thermo Fisher Scientific, Carlsbad, CA, USA). RT-qPCR was performed on a Viia7 (Life Technologies, Carlsbad, CA, USA) RT-qPCR system using PowerUp Sybr Green Master Mix (Life Technologies, Carlsbad, CA, USA) and 500 nM forward and reverse primers. Cycling parameters were 1 cycle of 95 °C for 15 sec, 95 °C for 5 sec and 56 °C for 20 sec for 40 cycles, and a melting curve measurement of 5 sec 0.5 °C incremental temperature increases from 60 to 95 °C. Primer sequences are listed in Table S1.

*IBA1 Immunohistochemistry*

For the chromogenic detection of IBA-1 positive cells, the right hemisphere of the brain was fixed in 4% paraformaldehyde for 2 days and cryoprotected in 30% sucrose. Coronal sections (40 µm) were collected using a freezing stage microtome (Microm HM 450, Thermo Scientific, Waltham, MA). Free-floating sections were treated with 1% hydrogen peroxide, washed three times for 10 minutes with phosphate buffered saline (PBS), incubated 1H in blocking solution (PBS containing 1% bovine serum albumin, 4% goat serum, 0.4% Triton X-100) and incubated overnight at 4 ^o^C with 1:1000 primary IBA-1antibodies diluted in DAKO antibody diluent (Agilent, Santa Clara, CA). Sections were then washed three times in PBS, incubated with biotinylated anti-rabbit antibody for 1H, washed three times in PBS, and incubated with Vectastain ABC Kit (Vector Laboratories, Burlingame, CA) reagents according to manufacturer’s instructions. Staining was visualized using 3,3’-diaminobenzidine (DAB) and urea-hydrogen peroxide tablets (Sigma-Aldrich, St. Louis, MO). Representative images were captured with a Leica DM2500 microscope (Leica Microsystems, Buffalo Grove, IL).

Using the fluorescent detection of IBA-1 positive cells, 40 µm sections were also used for the immunofluorescent assessment of microglia morphology. Here, free-floating sections were first washed for 10 min in 0.1% triton X-100 in phosphate buffered saline (PBST) at room temperature. Antigen retrieval was performed by incubating sections in 10 mM sodium citrate (pH = 6.0) with 0.5% Tween20 at 85°C for 15 min and then placing at room temperature for 30 min, as previously described ^24^. Sections were then blocked in 5% normal goat serum (NGS) in PBST for 1 h at room temperature. Samples were transferred into primary antibody solution, consisting of primary antibodies and blocking solution, at 4°C overnight. The primary antibody used were polyclonal rabbit anti-ionized binding adaptor molecule-1 (Iba-1, 1:1000) antibody. Slices were washed 3 times in PBST for 10 min, then incubated in blocking solution containing secondary antibodies for 1 h, protected from light. The secondary antibodies used were Alexa Fluor 488 goat anti-rabbit IgG (Thermo Fisher Scientific). Slices were washed 3 times in PBST for 10 min, then incubated in DAPI for 5 min. Slices were washed 3 times in PBS. Images were acquired using a Nikon A1R Confocal microscope (Nikon Instruments, Melville, NY).

***Gulf War Veteran Serum Samples***

Serum samples from 80 Gulf War veterans (40 GWI cases, 40 healthy GW veteran controls) from the DOD funded Boston Gulf War Illness Consortium (GWIC) biorepository were used for this study (Sullivan, PI). The GWIC biorepository study sites were IRB approved by Boston University and NOVA Southeastern University and the DOD Human Subjects Protection Office (HRPO). Samples from the GWI biorepository at the Boston and Miami sites were collected using the same written standard operating procedures for performing phlebotomy, plasma separation and aliquoting. All samples are stored at -80 °C. Gulf War veteran participants all provided written consent to participate in the study and to share biorepository samples for future relevant studies. The Kansas GWI criteria was used to determine GWI case and control status ^25^. The participants were administered a general demographic information and medical conditions questionnaire, the Kansas Gulf War and Health Questionnaire and Kansas Gulf War Experiences and Exposure Questionnaire, and the Structured Neurotoxicant Assessment Checklist (SNAC) as discussed in Janulewicz, et al ^26^. In this study, we analyzed the Kansas Gulf War and Health Questionnaire self-reported exposures to pyridostigmine bromide pill by asking whether or not they took the pyridostigmine bromide pills during the war. . All participants provided written informed consent to participate in the study. This study was reviewed and approved by the Boston University and Indiana University institutional review board.

The Kansas GWI criteria require that GW veterans endorse symptoms in at least 3 out of 6 symptom domains (fatigue/sleep problems, pain, cognitive, mood symptoms, gastrointestinal symptoms, respiratory symptoms and skin abnormalities). Controls were veterans from the 1990-1991 GW who did not endorse 3 out of 6 symptom domains associated with the Kansas criteria. Study participants were excluded if they reported being diagnosed with another medical condition that could explain the above mentioned symptoms^25^. GWIC study participants have an average age of 52 years, 15 years of education, 75% Caucasian and include 15% women^26^. Demographics are not significantly different between cases and controls. This study included serum samples from 40 veterans with GWI and 40 healthy veteran controls from the GWIC repository. For this study, both control GW veterans (n = 40) and veterans with GWI (n = 40) were well matched for age (mean age in controls = 54.4 and GWI = 51.1), Sex (controls = 10% women; Cases = 20% women), education (mean years in controls = 15 years and mean years in GWI = 15 years) and ethnic background (controls = 80% Caucasian, 12.5% African American, 8% other ethnicity and GWI cases = 68% Caucasian; 25% African American and 7.5% Other ethnicity).

**SUPPLEMENTAL REFERENCES**

1. Taetzsch T *et al.* Redox regulation of NF-kappaB p50 and M1 polarization in microglia. *Glia* 2015; **63**(3)**:** 423-440.

2. Catorce MN, Gevorkian G. LPS-induced Murine Neuroinflammation Model: Main Features and Suitability for Pre-clinical Assessment of Nutraceuticals. *Curr Neuropharmacol* 2016; **14**(2)**:** 155-164.

3. Qin L *et al.* Systemic LPS causes chronic neuroinflammation and progressive neurodegeneration. *Glia* 2007; **55**(5)**:** 453-462.

4. Qin L, Liu Y, Hong JS, Crews FT. NADPH oxidase and aging drive microglial activation, oxidative stress, and dopaminergic neurodegeneration following systemic LPS administration. *Glia* 2013; **61**(6)**:** 855-868.

5. Cooper CM *et al.* Memory and functional brain differences in a national sample of US veterans with Gulf War Illness. *Psychiat Res-Neuroim* 2016; **250:** 33-41.

6. Menon PM, Nasrallah HA, Reeves RR, Ali JA. Hippocampal dysfunction in Gulf War Syndrome. A proton MR spectroscopy study. *Brain Res* 2004; **1009**(1-2)**:** 189-194.

7. Engdahl BE *et al.* A Magnetoencephalographic (MEG) Study of Gulf War Illness (GWI). *Ebiomedicine* 2016; **12:** 127-132.

8. Odegard TN *et al.* Memory impairment exhibited by veterans with Gulf War Illness. *Neurocase* 2013; **19**(4)**:** 316-327.

9. Smylie AL *et al.* A comparison of sex-specific immune signatures in Gulf War illness and chronic fatigue syndrome. *BMC Immunol* 2013; **14:** 29.

10. Broderick G *et al.* A pilot study of immune network remodeling under challenge in Gulf War Illness. *Brain Behav Immun* 2011; **25**(2)**:** 302-313.

11. Vojdani A, Thrasher JD. Cellular and humoral immune abnormalities in Gulf War veterans. *Environ Health Perspect* 2004; **112**(8)**:** 840-846.

12. Skowera A *et al.* Cellular immune activation in Gulf War veterans. *J Clin Immunol* 2004; **24**(1)**:** 66-73.

13. Whistler T *et al.* Impaired immune function in Gulf War Illness. *BMC Med Genomics* 2009; **2:** 12.

14. Johnson GJ *et al.* Blood Biomarkers of Chronic Inflammation in Gulf War Illness. *PLoS One* 2016; **11**(6)**:** e0157855.

15. Zhang Q *et al.* Changes in immune parameters seen in Gulf War veterans but not in civilians with chronic fatigue syndrome. *Clin Diagn Lab Immunol* 1999; **6**(1)**:** 6-13.

16. Broderick G *et al.* Exploring the diagnostic potential of immune biomarker coexpression in Gulf War Illness. *Methods Mol Biol* 2012; **934:** 145-164.

17. Maloney SR, Jensen S, Gil-Rivas V, Goolkasian P. Latent viral immune inflammatory response model for chronic multisymptom illness. *Med Hypotheses* 2013; **80**(3)**:** 220-229.

18. Khaiboullina SF *et al.* Cytokine expression provides clues to the pathophysiology of Gulf War illness and myalgic encephalomyelitis. *Cytokine* 2015; **72**(1)**:** 1-8.

19. Parkitny L, Middleton S, Baker K, Younger J. Evidence for abnormal cytokine expression in Gulf War Illness: A preliminary analysis of daily immune monitoring data. *BMC Immunol* 2015; **16:** 57.

20. Broderick G *et al.* Altered immune pathway activity under exercise challenge in Gulf War Illness: an exploratory analysis. *Brain Behav Immun* 2013; **28:** 159-169.

21. Georgopoulos AP *et al.* Reduced Human Leukocyte Antigen (HLA) Protection in Gulf War Illness (GWI). *EBioMedicine* 2016; **3:** 79-85.

22. Abou-Donia MB *et al.* Screening for novel central nervous system biomarkers in veterans with Gulf War Illness. *Neurotoxicol Teratol* 2017; **61:** 36-46.

23. Alshelh Z *et al.* In-vivo imaging of neuroinflammation in veterans with Gulf War illness. *Brain Behav Immun* 2020; **87:** 498-507.

24. Jay TR *et al.* Disease Progression-Dependent Effects of TREM2 Deficiency in a Mouse Model of Alzheimer's Disease. *J Neurosci* 2017; **37**(3)**:** 637-647.

25. Steele L. Prevalence and patterns of Gulf War illness in Kansas veterans: association of symptoms with characteristics of person, place, and time of military service. *Am J Epidemiol* 2000; **152**(10)**:** 992-1002.

26. Janulewicz P *et al.* The Multiple Hit Hypothesis for Gulf War Illness: Self-Reported Chemical/Biological Weapons Exposure and Mild Traumatic Brain Injury. *Brain Sci* 2018; **8**(11).
