# Supplementary material for: Circulating HMGB1 is elevated in veterans with Gulf War Illness and triggers the persistent pro-inflammatory microglia phenotype in male C57Bl/6J mice
Source: Transl Psychiatry. 2021 Jul 12;11:390. doi: 10.1038/s41398-021-01517-1 (PMC8275600; doi:10.1038/s41398-021-01517-1)
Supplement: Supplementary file 2 — Supplemental Figure Legends and Tables [file 41398_2021_1517_MOESM2_ESM.docx]

**SUPPLEMENTAL FIGURE LEGENDS**

**Supplementary Figure 1. Persistent Microglia Morphology Changes in the Hippocampus 7 Days after LPS Administration.** Male C57BL/6 mice received a single IP injection of LPS or saline and microglia morphology was assessed 7 days later. (A) Representative images taken at 20X are shown and the scale bar depicts 100 μm for the chromogenic detection of changes in microglia morphology in the sub granular layer of the dentate gyrus (DG). The number of hypertrophic IBA1+ microglia cells were counted in the (B) subgranular layer of the DG. Hypertrophic cell = volume > 500 μm^3^. Data are reported as the mean ± SEM. *p < 0.05 and **p< 0.01 when compared to control (n =3).

**Supplementary Figure 2. rHMGB1 Failed to Elevate *Tnf* mRNA Expression in the Spleen.** Male C57BL/6 mice received a single tail vein injection of rHMGB1 or vehicle and *Tnf* mRNA expression was assessed 3 H later in the spleen. mRNA levels were analyzed by qRT-PCR and normalized to *Gapdh* using the 2-ΔΔCT method. Data are reported as the mean ± SEM. *p < 0.05 and **p< 0.01 when compared to control (n= 8).

**Supplementary Figure 3. rHMGB1 Failed to Elevate *Hmgb1* mRNA Expression in the Hippocampus Liver and Spleen.** Male C57BL/6 mice received a single tail vein injection of rHMGB1 or vehicle and *Hmgb1* mRNA expression was assessed in the (A) hippocampus (B) liver and (C) spleen. mRNA levels were analyzed by qRT-PCR and normalized to *Gapdh* using the 2-ΔΔCT method. Data are reported as the mean ± SEM. *p < 0.05 and **p< 0.01 when compared to control (n= 8).

**Supplementary Figure 4. Brain *Hmgb1* mRNA Expression Fails to Upregulate 7 Days after LPS Administration.** Male C57BL/6 mice received a single IP injection of LPS or saline and *Hmgb1* mRNA expression was assessed 7 days later in the (A) cortex, (B) hippocampus, (C) midbrain, by qRT-PCR and normalized to *Gapdh* using the 2-ΔΔCT method. Data are reported as the mean ± SEM. *p < 0.05 and **p< 0.01 when compared to control (n= 6).

**Supplementary Figure 5.** **The Acute Pro-Inflammatory Microglia Transcriptome 3 H after LPS administration.** Male C57BL/6 mice received a single IP injection of LPS or saline and microglia were isolated from whole brains at 3 H. The nanoString Mouse Neuroinflammation Panel was used to determine differential mRNA expression. (A) Heatmap depicting the normalized data 3 H LPS treatment. Orange indicates high expression; blue indicates low expression. (B). A volcano plot depicting the differential mRNA expression in 3 H LPS vs Saline. Values shown are the log2 (fold change) ratios of LPS treatment with respect to the saline group plotted against the -log10 (p-value). The black dots denote the analyzed genes with p-value below the given False Discovery Rate (FDR) or p-value threshold and the orange dots indicate the statistically significant genes (n=3).

**SUPPLEMENTAL TABLES**

**Supplementary Table S1.**

| **Association of HMGB1 Levels with Self-Reported Pyridostigmine Bromide Pill Exposure Adjusted for Age and GWI Status** | | | | |
| --- | --- | --- | --- | --- |
| **Have you ever taken pyridostigmine bromine (anti-nerve agent) pills?** | **Estimate** | **Standard Error** | **p-value** |  |
| **NO** | 0.6253 | 0.6055 | ref |  |
| **NOT SURE** | 0.7679 | 0.7238 | 0.8803 |  |
| **YES** | 1.9541 | 0.3102 | 0.0530 | |

*Statistical trend p= 0.530. Higher levels of serum HMGB1 in veterans are associated with reported Pyridostigmine Bromide use.

**Table S2. Genes Differentially Expressed in Microglia 3H After LPS: Acute Response**

| **Table S2 Genes Differentially Expressed in Microglia 3H After LPS: Acute Response** | | | | | |
| --- | --- | --- | --- | --- | --- |
| **mRNA** | **Up/ Down regulated** | **Log2 fold change** | **std error (log2)** | **P-value** | **Pathways** |
| **Hdac6** | ↑ | 0.953 | 0.0451 | 2.97E-05 | Cellular Stress, Epigenetic Regulation, Notch |
| **Pik3r1** | ↑ | 0.517 | 0.0269 | 4.34E-05 | Adaptive Immune Response, Angiogenesis, Apoptosis, Autophagy, Carbohydrate Metabolism, Cytokine Signaling, Growth Factor Signaling, Innate Immune Response, Insulin Signaling, Lipid Metabolism |
| **Lamp1** | ↓ | -0.258 | 0.0155 | 7.65E-05 | Autophagy, Microglia Function |
| **Atg5** | ↓ | -0.442 | 0.0369 | 0.000278 | Autophagy, Cellular Stress, Innate Immune Response |
| **Pik3cg** | ↓ | -0.707 | 0.0382 | 5.02E-05 | Adaptive Immune Response, Apoptosis, Growth Factor Signaling, Innate Immune Response |
| **Ncor2** | ↓ | -0.757 | 0.0431 | 6.15E-05 | Epigenetic Regulation, Notch |
| **Setd1b** | ↓ | -0.928 | 0.0607 | 0.000107 | Epigenetic Regulation |
| **Tmem64** | ↓ | -1.06 | 0.0836 | 0.000223 | Microglia Function |
| **Rad1** | ↓ | -1.06 | 0.0872 | 0.000265 | Cell Cycle, DNA Damage |
| **Mef2c** | ↓ | -1.26 | 0.0984 | 0.000211 | Growth Factor Signaling, Innate Immune Response, Microglia Function |
| **Bid** | ↓ | -1.56 | 0.126 | 0.000247 | Apoptosis, DNA Damage, Innate Immune Response |
| **Rtn4rl1** | ↓ | -1.89 | 0.156 | 0.00027 | Microglia Function |
| **Trim47** | ↓ | -1.99 | 0.0421 | 1.21E-06 | Microglia Function |
| **Gpr34** | ↓ | -1.99 | 0.12 | 7.82E-05 | Microglia Function |
| **Dusp7** | ↓ | -2.15 | 0.18 | 0.00028 | Adaptive Immune Response, Angiogenesis, Cytokine Signaling, Growth Factor Signaling, Innate Immune Response, Insulin Signaling, Microglia Function |
| **Sesn1** | ↓ | -2.48 | 0.124 | 3.68E-05 | DNA Damage |
| **Sall1** | ↓ | -2.75 | 0.217 | 0.000223 | Epigenetic Regulation |

**Table S3. Undirected and Directed Global Significance Scores of 20 Pathways: 7 Days after LPS Administration.**

|  | **Differential expression LPS vs Saline** | |
| --- | --- | --- |
| **Gene Sets** | **Undirected GSS** | **Directed GSS** |
| Inflammatory Signaling | **6.441** | **6.264** |
| Lipid Metabolism | **5.963** | **5.948** |
| Microglia Function | **5.93** | **3.49** |
| Adaptive Immune Response | **5.82** | **5.55** |
| Cytokine Signaling | **5.717** | **4.518** |
| NF-kB | **5.239** | **2.479** |
| Insulin Signaling | 5.163 | 4.902 |
| Innate Immune Response | 5.091 | 4.801 |
| Astrocyte Function | 5.075 | 4.561 |
| Angiogenesis | 4.685 | 4.21 |
| Cellular Stress | 4.67 | 4.412 |
| Matrix Remodeling | 4.593 | -2.258 |
| Neurons and Neurotransmission | 4.205 | 3.377 |
| Growth Factor Signaling | 4.091 | 3.345 |
| Autophagy | 3.94 | 3.426 |
| Apoptosis | 3.539 | 2.964 |
| Cell Cycle | 3.068 | 1.916 |
| Carbohydrate Metabolism | 2.95 | -2.619 |
| DNA Damage | 2.409 | 0.883 |
| Epigenetic Regulation | 1.782 | 1.091 |

**Table S4. Persistent Pro-inflammatory Microglia Gene Expression**

| **Table S4. Changes in Persistently Pro-inflammatory Microglia Cell Gene Expression** | | | | | |
| --- | --- | --- | --- | --- | --- |
| **mRNA** | **Up/ Down regulated** | **Log2 fold change** | **std error (log2)** | **P-value** | **Pathways** |
| **Ncr1** | ↑ | 4.24 | 0.2 | 2.93E-05 | Adaptive Immune Response, Innate Immune Response |
| **Gzma** | ↑ | 4.12 | 0.288 | 0.00014 | Neurons and Neurotransmission |
| **Ccl5** | ↑ | 3.69 | 0.204 | 5.55E-05 | Cellular Stress, Cytokine Signaling, Inflammatory Signaling, Innate Immune Response, Microglia Function |
| **C3** | ↑ | 3.59 | 0.246 | 0.000128 | Adaptive Immune Response, Autophagy, Inflammatory Signaling, Innate Immune Response, Microglia Function |
| **Cd72** | ↑ | 2.55 | 0.0826 | 6.54E-06 | Adaptive Immune Response, Inflammatory Signaling |
| **Cxcl10** | ↑ | 2.48 | 0.171 | 0.000132 | Astrocyte Function, Cytokine Signaling, Inflammatory Signaling, Innate Immune Response, Microglia Function |
| **Cd69** | ↑ | 2.42 | 0.15 | 8.63E-05 | Adaptive Immune Response |
| **Il2rg** | ↑ | 2.36 | 0.0966 | 1.68E-05 | Adaptive Immune Response, Angiogenesis, Cytokine Signaling, Growth Factor Signaling, Inflammatory Signaling, Insulin Signaling |
| **Irak3** | ↑ | 1.75 | 0.0974 | 5.69E-05 | Apoptosis, Cytokine Signaling, Innate Immune Response |
| **Cp** | ↑ | 1.75 | 0.141 | 0.000242 | Astrocyte Function |
| **Apoe** | ↑ | 1.59 | 0.0762 | 3.14E-05 | Astrocyte Function, Cellular Stress, Lipid Metabolism, Microglia Function, Neurons and Neurotransmission |
| **Tlr2** | ↑ | 1.19 | 0.0924 | 0.000211 | Growth Factor Signaling, Innate Immune Response, Microglia Function |
| **Anapc15** | ↑ | 1.02 | 0.0762 | 0.000182 | Cell Cycle, Cellular Stress |
| **Grn** | ↑ | 0.626 | 0.0454 | 0.000161 | Microglia Function |
| **Sqstm1** | ↑ | 0.615 | 0.0442 | 0.000155 | Autophagy, Cytokine Signaling, Growth Factor Signaling |
| **Cd84** | ↑ | 0.378 | 0.0199 | 4.53E-05 |  |
| **Tnfrsf17** | ↓ | -1.15 | 0.0511 | 2.34E-05 | Cytokine Signaling, NF-kB |
