# Supplementary figures and images for: Circulating HMGB1 is elevated in veterans with Gulf War Illness and triggers the persistent pro-inflammatory microglia phenotype in male C57Bl/6J mice
Source: Transl Psychiatry. 2021 Jul 12;11:390. doi: 10.1038/s41398-021-01517-1 (PMC8275600; doi:10.1038/s41398-021-01517-1)

**A.**

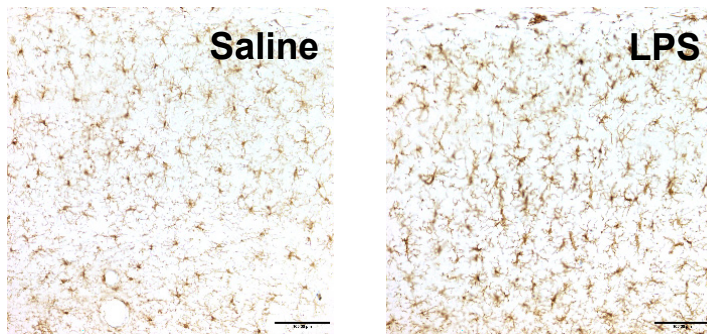

**B.**

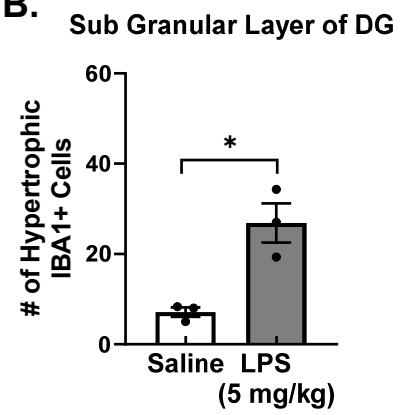

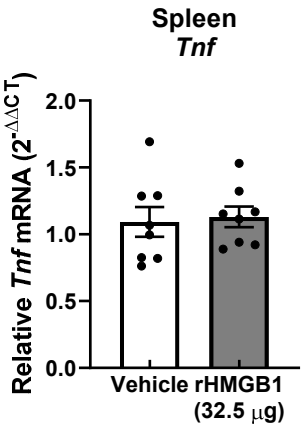

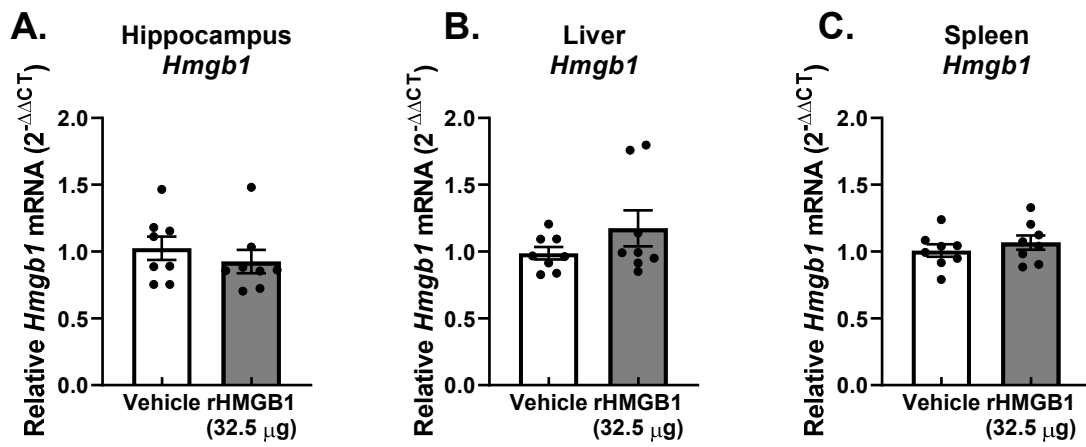

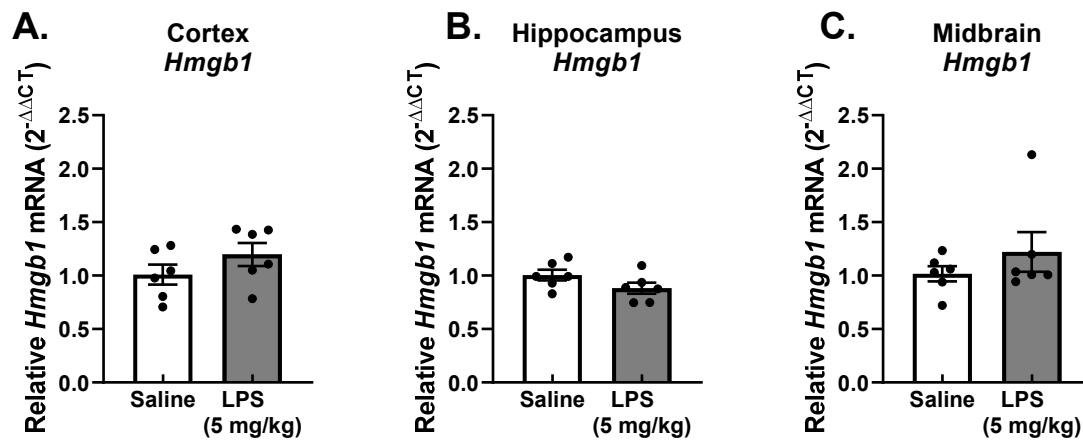

A.

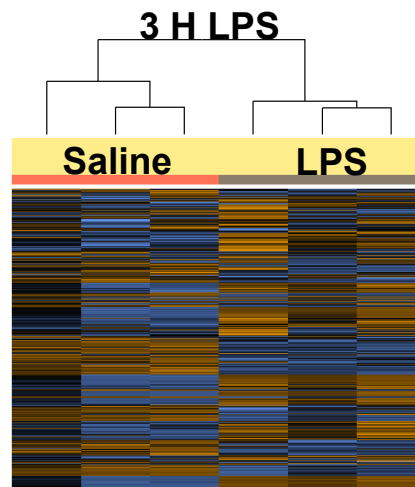

B.

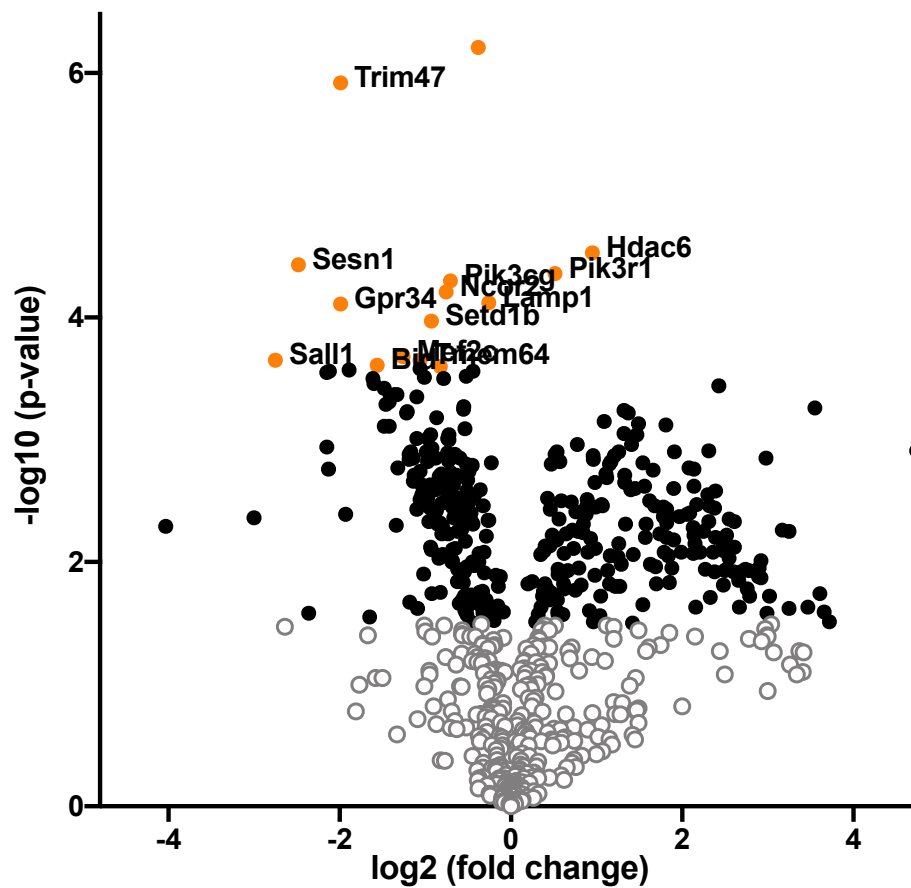

Supplement: Supplementary file 3 — Supplemental Figures [file 41398_2021_1517_MOESM3_ESM.pdf]
